# Screen-Based Sedentary Behaviors and Their Association With Metabolic Syndrome Components Among Adults in Mexico

**DOI:** 10.5888/pcd18.210041

**Published:** 2021-11-04

**Authors:** Nayeli Macías, Juan Espinosa-Montero, Eric Monterrubio-Flores, Lucía Hernández-Barrera, Catalina Medina-Garcia, Katia Gallegos-Carrillo, Ismael Campos-Nonato

**Affiliations:** 1 National Institute of Public Health, Center for Nutrition and Health Research, Cuernavaca, Morelos, México; 2 Mexican Institute of Social Security, Epidemiology and Health Services Research Unit, Mexico City, México

## Abstract

**Introduction:**

Approximately 25% of the adult population worldwide and 49.8% of Mexican adults have metabolic syndrome. Metabolic syndrome is the result of unhealthy dietary and sleeping patterns, sedentary behaviors, and physical inactivity. The objective of our study was to evaluate the association between sedentary behaviors as screen-based sedentary time (SBST) and each component of metabolic syndrome among adults who participated in the Mexico National Survey of Health and Nutrition Mid-way 2016.

**Methods:**

We analyzed sociodemographic, clinical, and physical activity data from 3,166 adults aged 20 years or older. The International Physical Activity Questionnaire was used to evaluate sedentary behavior. SBST was obtained by counting minutes per week spent watching television, playing video games, and interacting with computers and smartphones. We used Poisson regression to estimate the prevalence ratio of time in front of screens as a continuous variable and its association with metabolic syndrome.

**Results:**

The mean (SD) hours per day of SBST in men was 3.6 (0.4) and in women was 2.8 (0.2). The prevalence of metabolic syndrome was 59.6%. In men, the risk for metabolic syndrome increased 4% (*P* < .05) for each hour of SBST. Similarly, for each hour of SBST, the risk of abdominal obesity increased by 4% (*P* < .01). In women, we observed that the risk of hypertension or high-density lipoprotein cholesterol deficiency increased for each hour of SBST, and the risk of abdominal obesity increased for each hour of SBST in those who were inactive.

**Conclusion:**

Sedentary behavior based on screen time is associated with metabolic syndrome and its components among Mexicans, depending on hours of sleep. Current public health policies should consider strategies for reducing SBST.

**Summary**

**What is already known on this topic?**

Adults with sedentary activities and screen-based sedentary time (SBST) increase their risk of developing metabolic syndrome in the long term. In Mexico, no studies evaluate the association between sedentary behaviors, specifically SBST, and metabolic syndrome by using nationally representative information.

**What is added by this report?**

Our results contribute to the knowledge that may guide the design of interventions and initiatives that promote physical activity, with emphasis on reducing the time spent on screen-based sedentary behaviors and reducing the prevalence of metabolic syndrome and its components.

**What are the implications for public health practice?**

Current public health policies should consider strategies for reducing SBST among the population to fight the epidemic of metabolic syndrome in Mexico.

## Introduction

Metabolic syndrome is a cluster of at least 3 metabolic risk factors, including abdominal obesity, dyslipidemia, high blood pressure, and elevated fasting blood glucose (1). The syndrome is the result of unhealthy dietary patterns (2), inadequate sleep (3), sedentary behaviors, and physical inactivity (4). Approximately 25% of the adult population worldwide and 49.8% of adults in Mexico have metabolic syndrome (5).

Sedentary behaviors are independent and modifiable risk factors of metabolic syndrome (4) and include activities such as lying down and screen-based sedentary time (SBST). SBST includes watching television, computer use, and other forms of on-screen activities.

SBST during leisure time and work time has been associated with high waist circumference, high blood pressure, high fasting blood glucose, and high triglycerides clustered together as metabolic syndrome (6). A meta-analysis showed that adults spending more than 2 hours in sedentary activities and SBST during leisure time increased their risk of developing metabolic syndrome in the long term (7).

Although there is no estimation of SBST prevalence in Mexican adults, around 58% has reported not to be physically active (8). Moreover, no studies explore the association between SBST and metabolic syndrome by using nationally representative data. Furthermore, information about other factors that modify the association, such as physical activity and sleep duration, is scarce. Exploring whether interactions exist that are associated with variables described in other populations, such as hours of sleep, is necessary (9). Among the factors that are related to SBST and that might modify their association with metabolic syndrome are time spent sleeping, physical activity, education, sex, occupation, and socioeconomic status (3,4,6,7). The purpose of our study was to evaluate the association between sedentary behaviors, their modifiers, and each component of metabolic syndrome in adults who participated in the Mexico National Survey of Health and Nutrition Mid-way 2016.

## Methods

We used data sets from the Mexican 2016 Halfway National Health and Nutrition Survey (ENSANUT-MC 2016), a probabilistic survey with national, regional, urban, and rural strata representativeness, to perform this study. The main objective of ENSANUT-MC 2016 was to describe the health and nutrition status as well as determinants of the Mexican population. Details of the design, sampling size calculation, and methodology of survey administration have been described elsewhere (10). Data collection took place from May through October 2016, and a total of 8,626 adults aged 20 years or older were selected. A subsample of 3,188 participants who fasted 8 or more hours with complete information on biomarkers associated with metabolic syndrome was analyzed. The Ethical and Research Commissions of the National Institute of Public Health in Mexico approved our study.

Questionnaires were administered to gather sociodemographic information, personal history of lifestyle, and chronic disease background. A socioeconomic status index was generated through principal component analyses, and household characteristics, goods, and services available were considered. Socioeconomic status index was categorized in tertiles (low, middle, high). A standardized questionnaire was applied to obtain sociodemographic data such as age, level of education (primary school or less, middle school, high school, university level), region (north, central, Mexico City), and area (rural, urban).

The short version of the International Physical Activity Questionnaire (IPAQ) (11) was used to evaluate the physical activity levels of the study sample. This questionnaire asks the total time spent per week on moderate, walking, and vigorous physical activity and total time spent sitting on a typical day. In addition, the questionnaire includes 2 questions about the time per day, during the weekdays and weekends, spent using a screen-based device (computer, television, smartphone) for work or recreation. Adults were classified as physically active if they met at least 150 minutes per week of moderate to vigorous physical activity (11).

SBST was obtained by counting the minutes per week spent watching television, playing video games, using a smart telephone, and interacting with a computer. The variable SBST was used as a continuous variable and as a categorical variable divided into 3 groups: less than 2 hours per day 2 to 4 hours per day, and more than 4 hours per day (12). Participants self-reported sleep duration according to the following recommendations of the National Sleep Foundation: 7 to 9 hours (reference category), less than 7 hours, and more than 9 hours (risk categories) (13).

We calculated the probability of metabolic syndrome and its components by time spent in screen-based sedentary behaviors and category of sleeping time. The probability of abdominal obesity in active and inactive women was also calculated on SBST by hours of sleeping.

Trained team members measured weight, height, and waist circumference by using internationally accepted protocols (14). Body mass index (weight in kilograms divided by the square of the height in meters) was calculated and categorized according to the World Health Organization (WHO) classification: normal body mass index (18.5–24.9), overweight (25.0–29.9), and obese (≥30.0) (14). Waist circumference was categorized in quintiles and used to define abdominal obesity as a waist circumference of 80 cm or more for women and 90 cm or more for men (1).

Blood pressure was measured by using a digital sphygmomanometer Omron HEM-907 XL (Omron), following the protocol recommended by the American Heart Association (15). Adults were classified with hypertension when they had a systolic BP of 130 mm Hg or higher or a diastolic BP of 85 mm Hg or higher, or a pharmacologic treatment was prescribed for elevated BP. Participants were classified as having metabolic syndrome according to the harmonized criteria (1).

Glucose, triglycerides, and high-density lipoprotein cholesterol (HDL-C) concentrations were evaluated in participants who had fasted for at least 8 hours. Elevated fasting glucose was defined as fasting glucose of 100 mg/dL or higher or the use of pharmacologic treatment to control glucose. Hypertriglyceridemia was defined as a triglyceride concentration of 150 mg/dL or more and hypoalphalipoproteinemia was defined as HDL-C level of less than 50 mg/dL in men and less than 40 mg/dL in women (1).

The prevalence and 95% CI or mean and SD were estimated to characterize the sample considering categories of age, sex, SBST, physical activity, sleeping hours, sleep quality self-perception, anthropometric variables, metabolic syndrome components, and sociodemographic variables. The prevalence of metabolic syndrome components by the SBST category was calculated for men and for women. We used Poisson regression to estimate the prevalence ratio of each metabolic syndrome component, for metabolic syndrome and its association with SBST as a continuous variable adjusting by covariables: age (years), waist (quintiles of waist measurement in centimeters), physical activity (active), sleeping (<7.0 h/d, 7.0–9.0 h/d, >9.0 h/d), socioeconomic status (low, medium, high), education (primary school or less, middle school, high school, university level). To maintain the representativeness of the sample nationwide and by strata, we included an expansion factor in the model’s specification. A general *P* < .05 value set significance. All analyses were performed by using the SVY module for survey designs in Stata version 14 (StataCorp, LLC).

We tested interactions between SBST and other variables related to outcomes of interest such as sleep time, physical activity, socioeconomic status, and education level. We used an α value of .10 to consider a significant interaction.

## Results

We analyzed a subsample of 3,166 Mexican women and men with complete information on physical activity, metabolic syndrome indicators, and other variables. Mean (SD) hours per day spent in SBST were 3.6 (0.4) in men and 2.9 (0.2) in women. The prevalence of metabolic syndrome was 59.6% (95% CI, 54.7%–64.3%); it was higher in women (62.8%; 95% CI, 56.8%–68.4%) than in men (56.3%; 95% CI, 48.3%–64.0%). The percentage of adults with more than 4 hours per day of SBST was 26.9% (95% CI, 22.4%–32.0%). The most frequent metabolic syndrome component was abdominal obesity (78.6%; 95% CI, 75.3%–81.6%), and it was higher in women (89.3%; 95% CI, 86.8%–91.4%) than in men (67.7%; 95% CI, 61.3%–73.5%). Next was hypoalphalipoproteinemia (75.1%; 95% CI, 70.9%–78.9%), with no differences between women and men (Table 1).

**Table 1:** Sociodemographic and Anthropometric Characteristics Among Adults (N = 3,166), Mexico National Survey of Health and Nutrition Mid-way 2016^a^

Characteristic	Total, % (95% CI)	Men, % (95% CI)	Women, % (95% CI)
**Total**	100.0	49.3 (44.7–53.9)	50.7 (46.1–55.3)
**Age group, y**			
20–39	48.7 (44.4–53.0)	50.9 (44.3–57.5)	46.6 (41.6–51.6)
40–59	35.7 (31.9–39.7)	32.7 (27.3–38.7)	38.6 (33.9–43.4)
≥60	15.6 (13.5–17.9)	16.3 (13.3–19.9)	14.9 (12.2–18.0)
**Area**			
Rural	23.4 (20.2–26.9)	23.3 (19.1–28.0)	23.5 (19.9–27.5)
Urban	76.6 (73.1–79.8)	76.7 (71.9–80.9)	76.5 (72.5–80.1)
**Socioeconomic status^b^**			
Low	20.8 (17.8–24.1)	20.9 (16.9–25.7)	20.6 (17.5–24.2)
Medium	30.5 (27.1–34.1)	32.0 (26.3–38.3)	29.0 (25.2–33.1)
High	48.8 (44.1–53.4)	47.1 (39.5–54.8)	50.4 (45.1–55.6)
**Educational level**			
Primary or less	36.1 (32.3–40.1)	35.5 (22.9–41.4)	36.6 (31.9–41.7)
Middle school or high school	28.9 (25.2–33.0)	27.2 (21.5–33.7)	30.7 (25.5–36.3)
University level^c^	35.0 (30.2–40.1)	37.4 (30.1–45.2)	32.7 (26.7–39.5)
**Region**			
North	26.1 (22.6–30.0)	25.4 (20.2–31.5)	26.8 (21.6–32.8)
Central Mexico and Mexico City	47.6 (42.9–52.3)	48.8 (41.3–56.4)	46.4 (41.0–51.9)
South	26.3 (22.7–30.3)	25.8 (21.1–31.1)	26.8 (22.6–31.4)
**Body mass index classification**			
Normal weight (18.5–24.9)	22.2 (19.7–24.9)	25.5 (21.3–30.3)	19.0 (16.1–22.2)
Overweight (25.0–29.9)	42.2 (37.8–46.6)	46.3 (39.4–53.3)	38.2 (33.1–43.6)
Obese (≥30.0)	35.0 (30.9–39.4)	27.8 (22.5–33.7)	42.0 (36.8–47.4)
**Waist circumference^d^, cm**			
Quintile 1	79.5 (78.8–80.2)	80.7 (79.9–81.6)	78.5 (77.5–79.6)
Quintile 2	88.9 (88.3–89.7)	89.3 (88.3–90.4)	88.6 (88.2–88.9)
Quintile 3	95.0 (94.8–95.3)	94.9 (94.6–95.4)	95.0 (94.7–95.3)
Quintile 4	101.0 (100.7–101.4)	101.4 (100.9–101.9)	100.6 (100.3–101.0)
Quintile 5	115.3 (114.1–116.6)	115.3 (113.8–116.8)	115.4 (113.8–116.9)
**Metabolic syndrome^e^**			
No	40.4 (35.7–45.3)	43.7 (36.0–51.7)	37.2 (31.6–43.2)
Yes	59.6 (54.7–64.3)	56.3 (48.3–64.0)	62.8 (56.8–68.4)
**Metabolic syndrome components**			
Abdominal obesity	78.6 (75.3–81.6)	67.7 (61.3–73.5)	89.3 (86.8–91.4)
Hyperglycemia	31.7 (28.3–35.2)	31.0 (25.4–37.2)	32.3 (28.3–36.6)
Hypertriglyceridemia	56.3 (51.6–60.8)	59.4 (51.4–66.8)	53.2 (47.7–58.7)
Hypoalphalipoproteinemia	75.1 (70.9–78.9)	71.2 (64.4–77.1)	78.9 (73.9–83.4)
Hypertension	34.9 (31.1–39.0)	38.4 (32.1– 45.1)	31.6 (27.3–36.3)
**Screen-based sedentary time^f^, h/d**			
≤2	47.4 (42.9–51.9)	40.0 (33.7–46.6)	54.5 (48.9–59.9)
2–4	25.7 (22.5–29.2)	28.4 (22.9–34.6)	23.1 (19.1–27.6)
>4	26.9 (22.4–32.0)	31.6 (23.9–40.4)	22.4 (17.9–27.6)
**Physical activity^g^**			
Inactive	13.7 (11.0–16.9)	12.4 (8.8–17.3)	14.9 (10.9–19.9)
Active	86.3 (83.1–88.9)	87.6 (82.7–91.2)	85.1 (80.1–89.1)
**Time sleeping, h/d**			
<7	29.1 (24.5–34.1)	33.6 (26.2–41.9)	24.7 (20.1–29.9)
7–9	65.9 (61.2–70.5)	63.0 (54.9–70.3)	68.9 (63.6–73.7)
>9	4.9 (3.8–6.4)	3.4 (2.3–4.9)	6.4 (4.5–9.1)

a Statistical estimators were adjusted by the design of complex surveys.

b Socioeconomic status score was obtained with principal components analysis (household characteristics, goods, and services available were considered) and expressed as tertiles. Tertile 1 refers to the lowest score or low socioeconomic status; tertile 2 refers to the middle score or medium socioeconomic status; and tertile 3 is the category with the highest score or highest socioeconomic status.

c University comprises college and bachelor studies.

d For men, quintile 1 was 66.6–85.4; quintile 2, 85.5–91.9; quintile 3, 92.0–97.4; quintile 4, 97.5–105.0; quintile 5, 105.1–172.3. For women quintile 1, 50.5–85.4; quintile 2, 85.5–91.9; quintile 3, 92.0–97.4; quintile 4, 97.5–105.0; quintile 5, 105.1–167.4. Quintile 1 of waist circumference is the reference for men and women.

e Harmonizing metabolic syndrome, defined as having 3 or more of the following: waist circumference of ≥90 cm in men or ≥80 cm in women; triglycerides of ≥150 mg/dL or received pharmacologic treatment for hypertriglyceridemia; high-density lipoprotein cholesterol of <40 mg/dL in men or <50 mg/dL in women; systolic blood pressure of ≥130 mm Hg, diastolic blood pressure of ≥85 mm Hg, or a medical diagnosis of hypertension; fasting glucose of ≥100 mg/dL or previously diagnosed diabetes.

f Screen-based sedentary time was obtained by counting hours per week spent watching television, playing video games, and using a computer or smart telephone.

g Categorized according to World Health Organization recommendations, inactive defined as <150 min/wk or active defined as ≥150 min/wk of moderate to vigorous physical activity.

Men with SBST of more than 4 hours per day had a prevalence of metabolic syndrome of 55.6% (95% CI, 36.1%–75.1%), abdominal obesity of 80.1% (95% CI, 70.4%–89.7%), and hypoalphalipoproteinemia of 83.1% (95% CI, 73.3%–93.0%). Women with SBST of more than 4 hours per day had a prevalence of metabolic syndrome of 60.3% (95% CI, 44.8%–75.5%), abdominal obesity of 88.7% (95% CI, 82.6%–94.8%), and hypoalphalipoproteinemia of 89.1% (95% CI, 82.6%–95.7%). Because interaction between sexes and screen time was found, we evaluated separate models for men and for women (*P* < .10) (Table 2). In men, the risk for metabolic syndrome and abdominal obesity increased by 4% for each hour of SBST in those who slept less than 7 hours, and for those who slept more than 9 hours by 29% (Table 3).

**Table 2:** Prevalence of Metabolic Syndrome Components by Total Screen-Based Sedentary Time Among Adult Men and Women (N = 3,166), Mexico National Survey of Health and Nutrition Mid-way 2016

Screen-Based Sedentary Time^a^	Metabolic Syndrome,^b^ % (95% CI)	Hyperglycemia, % (95% CI)	Hypertriglyceridemia, % (95% CI)	Hypertension, % (95% CI)	Abdominal Obesity, % (95% CI)	Hypoalphalipoproteinemia, % (95% CI)
**Men, h/wk**						
≤2	52.5 (42.6–62.4)	36.4 (28.1–44.6)	64.5 (56.3–72.7)	38.5 (29.8–47.2)	57.6 (47.2–68.0)	66.6 (55.8–77.4)
2–4	57.3 (47.7–66.9)	28.5 (19.4–37.7)	60.1 (50.5–69.6)	34.9 (25.9–44.1)	65.1 (55.6–74.7)	63.4 (53.0–73.7)
>4	55.6 (36.1–75.1)	20.9 (10.2–31.5)	58.5 (38.6–78.5)	32.9 (18.7–46.9)	80.1 (70.4–89.7)	83.1 (73.3–93.0)
**Women, h/wk**						
≤2	67.6 (61.0–74.1)	35.4 (29.5–41.4)	58.8 (52.6–65.1)	32.1 (26.6–37.6)	90.9 (87.8–94.2)	77.7 (70.6–84.7)
2–4	54.3 (42.6–65.9)	25.0 (17.3–32.7)	51.2 (40.3–62.2)	23.2 (15.3–31.1)	87.7 (82.8–92.6)	74.4 (61.3–87.4)
>4	60.3 (44.8–75.8)	21.1 (12.6–29.5)	45.8 (30.3–61.3)	30.7 (14.8–46.6)	88.7 (82.6–94.8)	89.1 (82.6–95.7)

a Screen-based sedentary time was obtained by counting hours per week spent watching television, playing video games, and using a computer or smart telephone.

b Harmonizing metabolic syndrome, defined as having 3 or more of the following: a waist circumference of ≥90 cm in men or ≥80 cm in women; triglycerides of ≥150 mg/dL or received pharmacologic treatment for hypertriglyceridemia; high-density lipoprotein cholesterol of <40 mg/dL in men or <50 mg/dL in women; systolic blood pressure of ≥130 mm Hg, diastolic blood pressure of ≥85 mm Hg, or a medical diagnosis of hypertension; fasting glucose of ≥100 mg/dL or previously diagnosed diabetes.

**Table 3:** Association Between Sedentary Behavior and Metabolic Syndrome^a^ Components Among Men and Women, Mexico National Survey of Health and Nutrition Mid-way 2016

Characteristic	Metabolic Syndrome,^a^ PR (95% CI)	Hyperglicemia, PR (95% CI)	Hypertriglyceridemia, PR (95% CI)	Hypertension, PR (95% CI)	Abdominal Obesity, PR (95% CI)	Hypoalphalipoproteinemia, PR (95% CI)
**Men**						
**Screen-based sedentary time, h/d**	1.04 (1.00–1.08)^b^	0.88 (0.82–0.95)^c^	0.94 (0.89–1.00)	0.94 (0.88–1.01)	1.04 (1.02–1.07)^c^	1.03 (1.01–1.05)^c^
**Age d, y**	1.01 (1.01–1.02)^c^	1.03 (1.01–1.04)^c^	0.99 (0.98–1.00)	1.01 (0.99–1.03)	1.01 (1.00–1.02)	1.00 (1.00–1.01)
**Waist e, cm**						
Quintile 2	—	1.48 (0.72–3.06)	1.55 (1.02–2.36)^b^	1.34 (0.68–2.64)	—	1.41 (1.13– 1.78)^c^
Quintile 3	—	1.90 (1.01–3.56)^b^	1.92 (1.34–2.76)^c^	1.59 (0.86–2.95)	—	1.34 (1.05–1.70)
Quintile 4	—	1.91 (0.98–3.73)	2.18 (1.58–3.02)^c^	2.24 (1.22–4.09)^b^	—	1.51 (1.21–1.88)^b^
Quintile 5	—	3.25 (1.76–6.03)^b^	2.20 (1.55–3.13)^c^	3.27 (1.93–5.54)^c^	—	1.32 (1.03–1.69)^b^
**Physical activity^f^**						
Active	0.71 (0.59–0.84)^c^	0.72 (0.51–1.01)	0.78 (0.66–0.93)^c^	0.80 (0.53–1.22)	0.86 (0.72–1.03)	0.95 (0.85–1.07)
**Time sleeping^g^, h/d**						
<7 h	1.42 (1.10–1.85)^c^	0.90 (0.67–1.20)	0.88 (0.72–1.08)	1.03 (0.78–1.37)	1.04 (0.90–1.21)	0.91 (0.78–1.07)
>9 h	1.12 (0.65–1.92)	0.94 (0.54–1.64)	0.96 (0.69–1.32)	0.95 (0.57–1.58)	1.29 (1.05–1.59)^b^	1.06 (0.92–1.21)
**Socioeconomic status^h^**						
Medium	1.01 (0.79–1.29)	0.80 (0.57–1.14)	1.07 (0.85–1.36)	0.99 (0.73–1.36)	1.06 (0.84–1.34)	0.99 (0.89–1.10)
High	1.38 (1.10–1.72)^c^	1.23 (0.87–1.74)	1.12 (0.90–1.41)	0.98 (0.67–1.42)	1.24 (1.01–1.51)	0.83 (0.71–0.98)
**Education^i^**						
Middle school	0.92 (0.72–1.18)	1.38 (0.97–1.97)	1.05 (0.86–1.30)	0.68 (0.48–0.96)^b^	0.98 (0.77–1.23)	1.02 (0.88–1.16)
University level	0.89 (0.69–1.15)	1.42 (0.93–2.17)	1.07 (0.85–1.36)	0.90 (0.65–1.25)	0.88 (0.72–1.09)	1.05 (0.89–1.22)
**Women**						
**Screen-based sedentary time, h/d**	1.01 (0.97–1.06)	0.94 (0.85–1.05)	0.97 (0.93–1.02)	1.07 (1.01–1.13)^b^	1.04 (1.02–1.05)^c^	1.03 (1.01–1.05)^c^
**Age d, y**	1.02 (1.02–1.03)^c^	1.03 (1.02–1.05)^c^	1.0 (1.0–1.10)^c^	1.04 (1.03–1.06)^c^	1.00 (1.00–1.01)^c^	1.00 (0.99–1.01)
**Waist,^e^ cm**						
Quintile 2	—	1.26 (0.76–2.10)	1.60 (1.11–2.31)	1.73 (1.03–2.92)	—	1.42 (1.13–1.78)^c^
Quintile 3	—	1.67 (1.06–2.64)^b^	1.82 (1.20–2.76)^c^	1.35 (0.78–2.34)	—	1.34 (1.05–1.70)^b^
Quintile 4	—	2.51 (1.61–3.91)^c^	2.22 (1.58–3.14)^c^	1.77 (1.12–2.79)^b^	—	1.51 (1.22–1.88)^c^
Quintile 5	—	2.74 (1.74–4.32)^c^	1.91 (1.32–2.80)^c^	2.84 (1.73–4.66)^c^	—	1.32 (1.04–1.69)^b^
**Physical activity^f^**						
Active	1.15 (0.92–1.43)	1.07 (0.81–1.41)	1.17 (0.90–1.51)	1.20 (0.88–1.64)	1.09 (1.00–1.18)	0.9 (0.8–1.1)
**Time sleeping^g^, h/d**						
<7 hours	0.94 (0.73–1.21)	1.06 (0.76–1.48)	0.88 (0.71–1.09)	0.87 (0.58–1.32)	1.08 (1.02–1.15)^c^	0.92 (0.79–1.08)
>9 hours	1.01 (0.78–1.30)	0.32 (0.15–0.66)	1.12 (0.86–1.44)	1.21 (0.74–1.98)	1.08 (1.03–1.14)^c^	1.06 (0.93–1.21)
**Socioeconomic status^h^**						
Medium	1.08 (0.91–1.28)	0.8 (0.6–1.1)	0.9 (0.8–1.2)	1.0 (0.7–1.5)	1.0 (0.9–1.1)	0.9 (0.8–1.1)
High	0.86 (0.68–1.09)	0.4 (0.3–0.6)	0.8 (0.6–1.2)	0.9 (0.6–1.5)	1.0 (0.9–1.1)	0.8 (0.7–0.9)
**Education^i^**						
Middle school	0.9 (0.7–1.2)	1.2 (0.9–1.6)	1.0 (0.8–1.4)	0.8 (0.5–1.2)	0.9 (0.9–1.0)	1.0 (0.8–1.2)
University level	0.9 (0.7–1.2)	1.3 (0.9–1.8)	1.2 (0.9–1.5)	0.6 (0.4–0.9)	0.8 (0.8–0.9)	1.0 (0.9–1.2)

Abbreviations: —, indicates the absence of value, depending on the variable defined as outcome; PR, prevalence ratio.

a Harmonizing metabolic syndrome, defined as having 3 or more of the following: a waist circumference of ≥90 cm in men or ≥80 cm in women; triglycerides of ≥150 mg/dL or received pharmacologic treatment for hypertriglyceridemia; high-density lipoprotein cholesterol of <40 mg/dL in men or <50 mg/dL in women; systolic blood pressure of ≥130 mm Hg, diastolic blood pressure of ≥85 mm Hg, or a medical diagnosis of hypertension; fasting glucose of ≥100 mg/dL or previously diagnosed diabetes.

b *P* < .05.

c *P* < .01.

d Age as a continuous variable, by each unit of age, the possibility of presenting metabolic syndrome increases an average of 8.0%.

e For men quintile 1, 66.6–85.5; quintile 2, 86.0–91.9; quintile 3, 92.0–97.5; quintile 4, 97.5–105.0; quintile 5, 105.1–172.3. For women quintile 1, 50.5–85.5; quintile 2, 85.5–91.9; quintile 3, 92.0–97.5; quintile 4, 97.5–105.1; quintile 5, 105.1–167.4. Quintile 1 of waist circumference is the reference for men and women.

f Categorized according to World Health Organization recommendations, inactive is defined as <150 min/wk or active defined as ≥150 min/wk of moderate to vigorous physical activity. Inactive is the reference.

g Reference is 7 to 9 hours.

h Socioeconomic status score was obtained with principal components analysis (household characteristics, goods, and services available were considered) and expressed as tertiles. Tertile 1 refers to the lowest score or low socioeconomic status; tertile 2 refers to the middle score or medium socioeconomic status; and tertile 3 is the category with the highest score or highest socioeconomic status. Low is the reference.

i Level of education: primary school (reference category in the model), middle school includes high school and middle school; university level comprises college and bachelor studies. School information was self-reported in sociodemographic questionnaire.

**Figure 1:**
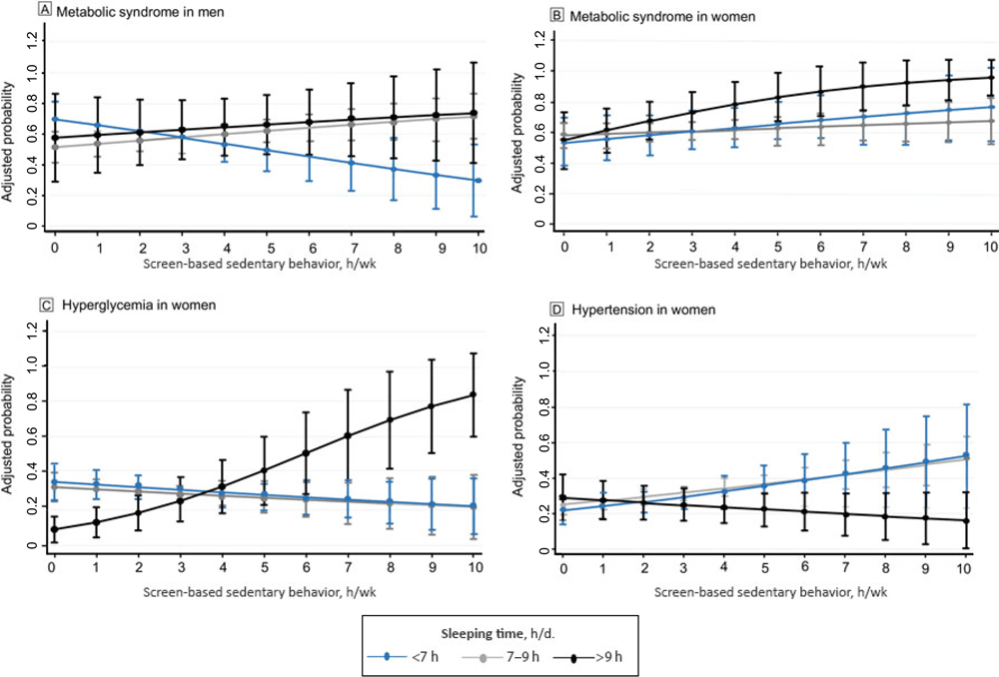
Screen-based sedentary behaviors and adjusted probability of metabolic syndrome in men and metabolic syndrome, hyperglycemia, and hypertension in women for every hour in screen-based sedentary time, Mexico National Survey of Health and Nutrition Mid-way 2016.

**Figure 2:**
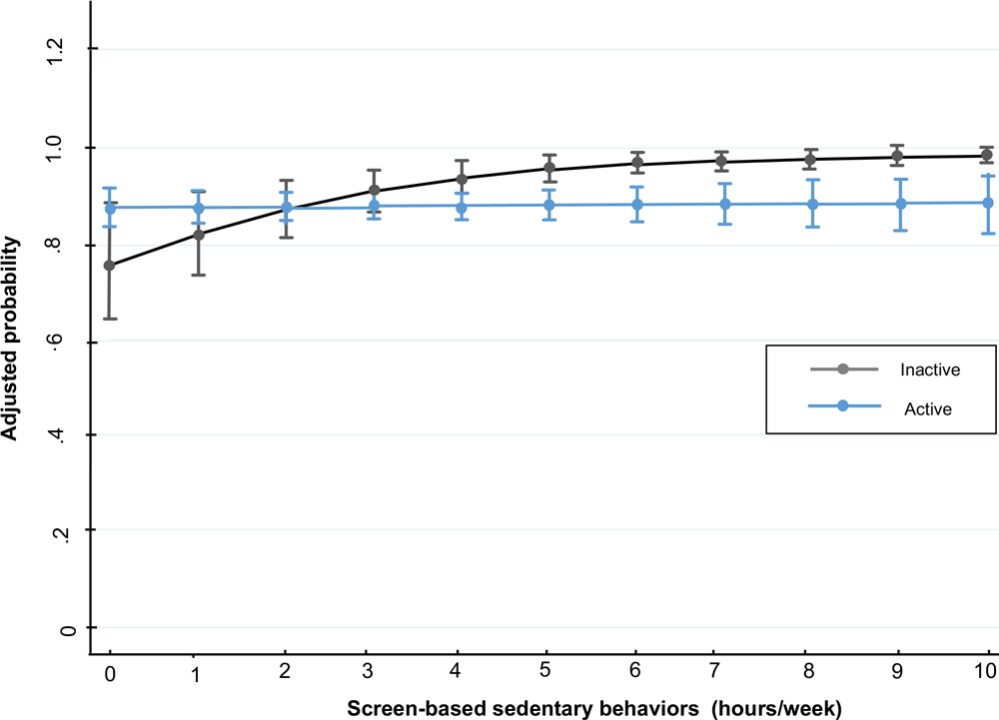
Screen-based sedentary behaviors and adjusted probability of abdominal obesity in women, Mexico National Survey of Health and Nutrition Mid-way 2016.

For men who slept 7 to 9 hours per day, for every hour in SBST, the probability (PR) of having metabolic syndrome increased 4% (PR, 1.04; 95% CI, 1.00–1.08; *P* < .05) (Figure 1). For women who slept longer than 9 hours per day, for every hour in SBST the probability of having metabolic syndrome (PR, 1.08; 95% CI, 1.02–1.14; *P* < .01) and hyperglycemia (PR, 1.29; 95% CI, 1.15–1.44; *P* < .001) increased. Moreover, the probability of having hypertension increased (PR, 1.07 [1.01–1.13; *P* < .05) in women who slept 7 to 9 hours for every hour invested in SBST (Figure 1). In inactive women, the probability of having abdominal obesity increased (PR, 1.04; 95% CI, 1.02–1.05; *P* < .001) by every hour spent in SBST (Figure 2).

## Discussion

In this nationwide representative sample, we found an association between SBST and metabolic syndrome that was modified by sleep duration per night in men. Among women who slept more than 9 hours per night, SBST increased the probability of hyperglycemia and metabolic syndrome in women. Furthermore, inactive women had an increased likelihood of abdominal obesity for every hour they spent in SBST.

Differences by sex have been reported related to physical activity and sedentary behaviors (9). Those differences might explain the contrasting associations among populations with health outcomes as metabolic syndrome components (16). However, we found no significant differences in the prevalence of metabolic syndrome between men and women like we did for its components. This is consistent with what has been observed in other studies for metabolic syndrome components (9,17).

We found that SBST was associated with metabolic syndrome interaction with hours of sleep; to our knowledge, evidence of this interaction is scarcely ever explored (9,18). Chaput et al (19) found in a longitudinal study that adults who slept a short time (<6 h/d) or a long time (>9 h/d) gained more visceral fat than those sleeping 7 to 8 hours per night. Some authors have found that sleep duration is related to glucose and blood pressure dysregulation through hormonal changes as increased catecholamine production and insulin function is impaired (3). This mechanism combined with body fat increase attributable to sedentary behaviors such as screen-based activities and sleeping patterns might explain the association of SBST and sleep with metabolic syndrome and its components found in our study.

Highly sedentary individuals and individuals with a high level of screen time are more likely to have metabolic syndrome. The time spent in sedentary behaviors can be related to the duration of sleep, as found in observational studies (19,20). Another possible explanation for the SBST and sleeping time interaction is that more hours of sleeping and screen time may be replacing time than can be used in moderate or vigorous physical activities that have been negatively related to metabolic syndrome.

In our study, women who slept more than 9 hours increased their risk of metabolic syndrome and hyperglycemia for each hour spent in SBST (9). Women who slept less than 7 hours showed a nonsignificant but suggested higher risk of metabolic syndrome and hypertension for every hour they spent in SBST. This tendency must be confirmed with cohort studies. Our findings about sleep duration and metabolic syndrome may be explained by the U-shaped association between these variables, regardless of potential confounders (21).

For each hour of SBST, men who slept 7 to 9 hours a day increased their risk of metabolic syndrome. Xiao et al found a similar tendency for sitting time; however, they did not evaluate the interaction between sedentary behavior and sleeping time like we did (9).

Contrary to our expectations, in men, the risk of hyperglycemia decreased for each hour spent in SBST. A nonsignificant negative association between SBST and hyperglycemia has been reported before (22). Part of the explanation for this may be overreporting or underreporting of time spent in recreational activities or screen time, observed when SBST is not measured with more accurate instruments such as accelerometers.

We did not find an interaction between abdominal obesity and SBST, either in men or in women. More studies that explore the association between SBST and abdominal obesity and its influence on metabolic outcomes are needed. However, we found that physically inactive women increased their risk of abdominal obesity for each hour spent in SBST. This is consistent with results of a study in China where women who sit for a longer time than active women were more likely to have abdominal obesity (9). Physical activity has a preventive effect against metabolic syndrome components and may be independently associated with sedentary behaviors. However, we did not find an association between SBST and hypertriglyceridemia, hyperglycemia, or hypertension modified by physical activity.

The mechanism behind sedentary behavior and metabolic syndrome may imply less muscle activity and energy expenditure. Also, low blood flow and increased stress occur from vascular shear. These changes in vascular endothelial functions are likely to promote alterations in homeostasis (23). Another plausible explanation is that the association between SBST and metabolic syndrome may be due to the lower rate of glucose elimination in skeletal muscle attended by less suppression of glucose production in the liver, suppression of lipolysis, and mitochondrial dysfunction of skeletal muscle. All of this leads to the accumulation of ectopic lipids and the generation of multiorgan metabolic dysfunction (24). Additionally, SBST decreases the activity of the lipoprotein lipase enzyme in the capillary endothelium and consequently diminishes the concentrations of HDL-C and increases the concentration of triglycerides.

Imprecision in quantifying sedentary behavior through questionnaires that measure sedentary activities, has been recognized (25). This contributes to the generation of measurement biases and misclassification of SBST. These biases and the underestimation of SBST likely contributed to the fact that we found no association between SBST and hypertension. In our study, exposure to SBST was less than 30 hours per week, which is less than the average reported in the category of risk in other studies (>42 h/wk), where they did find an association (9). Lack of sufficient exposure may explain no association between SBST and hypertension in our study.

The main limitation in our study is its cross-sectional design, which does not allow establishing causal associations. No consensus exists on which is the best indicator to describe sedentary behavior; however, activities performed for long periods, such as watching television, being in front of screens, and sitting can be reliable (26). Possibly, confounding factors were not taken into account, or the questionnaires were not accurate enough to avoid a residual confounding effect that could affect our results. Despite this, we consider that the method for measuring SBST allows us to develop a robust estimator of sedentary behavior. The IPAQ is a subjective instrument to measure the time spent in sedentary behavior (27); however, the results it produces are similar to those generated by direct methods such as the accelerometer. SBST did include smartphone use as part of a category of other sedentary behaviors, which may underestimate the association between this behavior and outcomes of interest. Validation studies about smartphone use and its inclusion in our instruments are recommended.

Among our study strengths, we highlight that this research is representative at the national level and that standardized instruments and techniques to measure sedentary behaviors, biomarkers, and anthropometric indicators were used. Moreover, the error of anthropometric indicators must be minimal because all researchers who measured waist and height in the field were standardized until they accomplished a 95% reproducibility and accuracy. Therefore, although the possibility of measurement error existed, the effect did not change the orientation of the results or their interpretation.

Among Mexicans, sedentary behavior based on screen time is associated with metabolic syndrome and its components, depending on the number of hours of sleep. Our results contribute to the knowledge to guide the design of interventions and initiatives that promote physical activity with emphasis on reducing the time spent on screen-based sedentary behaviors and reducing the prevalence of metabolic syndrome and its components. Current public health policies should consider strategies for reducing SBST among the population as a measure to fight the epidemic of metabolic syndrome in Mexico.
